# Meta-analysis Reveals Genome-Wide Significance at 15q13 for Nonsyndromic Clefting of Both the Lip and the Palate, and Functional Analyses Implicate *GREM1* As a Plausible Causative Gene

**DOI:** 10.1371/journal.pgen.1005914

**Published:** 2016-03-11

**Authors:** Kerstin U. Ludwig, Syeda Tasnim Ahmed, Anne C. Böhmer, Nasim Bahram Sangani, Sheryil Varghese, Johanna Klamt, Hannah Schuenke, Pinar Gültepe, Andrea Hofmann, Michele Rubini, Khalid Ahmed Aldhorae, Regine P. Steegers-Theunissen, Augusto Rojas-Martinez, Rudolf Reiter, Guntram Borck, Michael Knapp, Mitsushiro Nakatomi, Daniel Graf, Elisabeth Mangold, Heiko Peters

**Affiliations:** 1 Institute of Human Genetics, University of Bonn, Bonn, Germany; 2 Department of Genomics, Life&Brain Center, University of Bonn, Bonn, Germany; 3 Institute of Genetic Medicine, Newcastle University, International Centre for Life, Newcastle upon Tyne, United Kingdom; 4 Department of Biomedical and Specialty Surgical Sciences, University of Ferrara, Italy; 5 Orthodontic Department, College of Dentistry, Thamar University, Thamar, Yemen; 6 Department of Obstetrics and Gynaecology, ErasmusMC, Rotterdam, Netherlands; 7 Department of Epidemiology, Radboud University Medical Center, Nijmegen, Netherlands; 8 Department of Biochemistry and Molecular Medicine, School of Medicine, and Centro de Investigación y Desarrollo en Ciencias de la Salud, Universidad Autonoma de Nuevo Leon, Monterrey, Mexico; 9 Department of Otolaryngology—Head and Neck Surgery, Section of Phoniatrics and Pedaudiology, University of Ulm, Ulm, Germany; 10 Institute of Human Genetics, University of Ulm, Ulm, Germany; 11 Institute of Medical Biometry, Informatics and Epidemiology, University of Bonn, Bonn, Germany; 12 Division of Anatomy, Kyushu Dental University, Kitakyushu, Japan; 13 Orofacial Development and Regeneration, Institute of Oral Biology, Center for Dental Medicine, University of Zurich, Zurich, Switzerland; 14 Departments of Dentistry and Medical Genetics, Faculty of Medicine and Dentistry, University of Alberta, Edmonton, Canada; University of Oxford, UNITED KINGDOM

## Abstract

Nonsyndromic orofacial clefts are common birth defects with multifactorial etiology. The most common type is cleft lip, which occurs with or without cleft palate (nsCLP and nsCLO, respectively). Although genetic components play an important role in nsCLP, the genetic factors that predispose to palate involvement are largely unknown. In this study, we carried out a meta-analysis on genetic and clinical data from three large cohorts and identified strong association between a region on chromosome 15q13 and nsCLP (*P* = 8.13×10^−14^ for rs1258763; relative risk (RR): 1.46, 95% confidence interval (CI): 1.32–1.61)) but not nsCLO (*P* = 0.27; RR: 1.09 (0.94–1.27)). The 5 kb region of strongest association maps downstream of *Gremlin-1* (*GREM1*), which encodes a secreted antagonist of the BMP4 pathway. We show during mouse embryogenesis, *Grem1* is expressed in the developing lip and soft palate but not in the hard palate. This is consistent with genotype-phenotype correlations between rs1258763 and a specific nsCLP subphenotype, since a more than two-fold increase in risk was observed in patients displaying clefts of both the lip and soft palate but who had an intact hard palate (RR: 3.76, CI: 1.47–9.61, *P*_diff_<0.05). While we did not find lip or palate defects in *Grem1*-deficient mice, wild type embryonic palatal shelves developed divergent shapes when cultured in the presence of ectopic Grem1 protein (*P* = 0.0014). The present study identified a non-coding region at 15q13 as the second, genome-wide significant locus specific for nsCLP, after 13q31. Moreover, our data suggest that the closely located *GREM1* gene contributes to a rare clinical nsCLP entity. This entity specifically involves abnormalities of the lip and soft palate, which develop at different time-points and in separate anatomical regions.

## Author Summary

Clefts of the lip and palate are common birth defects, and require long-term multidisciplinary management. Their etiology involves genetic factors and environmental influences and/or a combination of both, however, these interactions are poorly defined. Moreover, although clefts of the lip may or may not involve the palate, the determinants predisposing to specific subphenotypes are largely unknown. Here we demonstrate that variations in the non-coding region near the *GREM1* gene show a highly significant association with a particular phenotype in which cleft lip and cleft palate co-occur (nsCLP; *P* = 8.13×10^−14^). Our data suggest that the risk is even higher for patients who have a cleft lip and a cleft of the soft palate, but not of the hard palate. Interestingly, this subphenotype corresponds to the expression of the mouse *Grem1* gene, which is found in the developing lip and soft palate but not in the hard palate. While *Grem1*-deficient mice display no lip or palate defects, we demonstrate that ectopic Grem1 protein alters palatal shelve morphogenesis. Together, our results identify a region near *GREM1* as the second, genome-wide significant risk locus for nsCLP, and suggest that deregulated *GREM1* expression during craniofacial development may contribute to this common birth defect.

## Introduction

Nonsyndromic cleft lip with or without cleft palate (nsCL/P) is a common human birth defect with a multifactorial etiology, including a strong genetic component [1, 2]. Previous studies have identified 16 genetic risk loci for nsCL/P. These studies comprised candidate gene and linkage analyses [3–5], genome-wide association studies (GWAS) with follow-up approaches [6–11], and a meta-analysis [12]. Despite these advances in deciphering the genetic architecture of nsCL/P, a number of additional risk loci still await identification. Some of these as yet unknown susceptibility variants may be detectable in GWAS datasets but have escaped detection at a genome-wide significant level due to low statistical power, which is secondary to limited sample sizes. This suggests that further risk variants for nsCL/P might be identified via one of the following approaches: the combining of available data sets, targeted replication analyses in independent cohorts, and/or the reduction of clinical heterogeneity using detailed subphenotype information.

NsCL/P shows considerable phenotypic variability in terms of affected anatomical structures, and can be subdivided into two main forms: nonsyndromic cleft lip only (nsCLO) and clefts involving both the lip and the palate (nsCLP) [2]. This distinction is important in terms of the degree of physical handicap and treatment. Although epidemiological data indicate that these subtypes are determined at least in part by genetic predisposition [13], few data are available concerning the specific genetic factors determining the formation of nsCLP as opposed to nsCLO. To date, one locus (at 13q31) has shown a specific association with nsCLP but not with nsCLO [12, 14], while *IRF6* has shown a predominant effect in nsCLO [5].

Previous research has implicated the *Gremlin-1* (*GREM1*) locus in human orofacial clefting. This research has involved the investigation of gene networks and, more recently, the finding of association between variants at 15q13 and human nsCL/P [10, 15]. However, associations of common variants were not yet significant at the genome-wide level. Also, sequencing studies of *GREM1* in nsCL/P patients and controls have been conducted in limited sample sizes only, with inconclusive results: although our group has previously generated some evidence for the role of rare variants in the *GREM1* coding and untranslated region [16], the functional relevance of the identified variants remained unclear, and the results of burden analyses varied depending on the test applied. In another sequencing study, no deleterious rare variants were identified in *GREM1* [15].

Analyses of *Grem1*-deficient mouse models have shown that during embryogenesis *Grem1* function is crucial for limb development and kidney formation. However, complete loss of *Grem1* function causes no obvious craniofacial defects [17, 18]. GREM1 acts as a secreted antagonist of various members of the bone morphogenetic protein (BMP) family, which has been shown to play a critical role in both lip and palate development [19, 20]. Notably, previous research has indicated a particular role for BMP4, which is involved in facial genesis [21, 22]. Moreover, rare mutations within *BMP4* have been associated with human clefting [23], and it is established that soluble GREM1 binds with high affinity to BMP4 [24]. Loss-of-function and overexpression studies of the related Bmp antagonist Noggin in mice have previously demonstrated the critical role of restricted Bmp signaling during lip and palate development, as well as for midfacial morphogenesis [25–27].

The present study analyzed the putative risk locus on human chromosome 15q13 in different nsCL/P cohorts, using detailed clinical subphenotype information. The genetic analyses were complemented by functional analyses of the mouse ortholog of the *GREM1* gene, which is located adjacent to the associated region. Finally, existing comprehensive genomics data sets were analyzed to annotate the associated region and to establish *GREM1* as a plausible candidate gene for nsCL/P at 15q13.

## Results

### The 15q13 region is a genome-wide significant risk locus for nsCL/P

**Fig 1:**
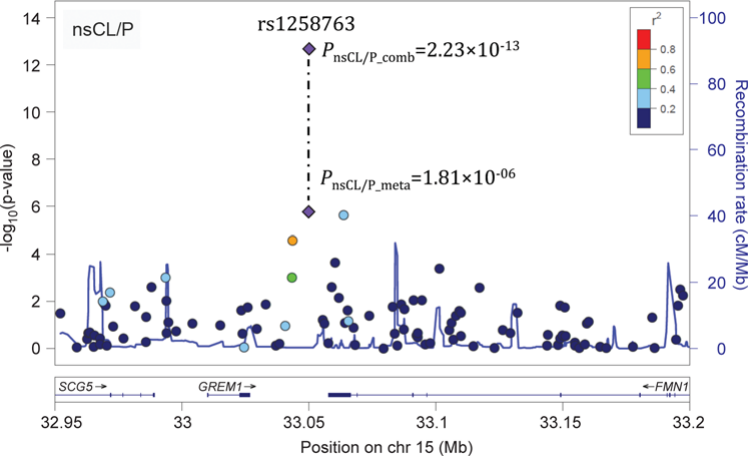
Regional association plot for the 15q13 region. *P*-values for SNPs at 15q13 that were analyzed as part of the Ludwig 2012 meta-analysis (*P*_nsCL/P_meta_) are plotted against their chromosomal position (hg19). Full data are provided in S1 Dataset. For each variant, color code denotes linkage disequilibrium to rs1258763, based on 1000genomes. After combination with data from replication I and II, the top variant rs1258763 (purple diamond; indicated by dotted line) reaches genome-wide significance. Plot was generated using LocusZoom [28].

Regional association statistics for 219 variants at 15q13 (chr15: 32.95–33.5 Mb, hg19) were extracted from a previously published meta-analysis (referred to as Ludwig 2012 meta-analysis) [12] (**Table 1**, **S1 Dataset**). The top associated variant at 15q13 in this Ludwig 2012 meta-analysis data set was rs1258763 (*P*_nsCL/P_*_*_meta_all_ = 1.81×10^−06^, **Fig 1** and **S1 Dataset**), with the A-allele representing the risk allele.

**Table 1:** Sample overview.

Study cohort ^a^	Design	Ethnicity	Sample size ^c^	Subphenotype information available?	References ^d^	
					Sample description	Genotypes
				
Ludwig 2012 meta-analysis ^b^	case-control	Central European ^e^	399 cases, 1,318 controls	yes	[29]	[10], [12] ^f^
	trio	European	666 trios	yes	[6]	[12]
		Asian	795 trios			
replication I	case-control	Central European ^e^	223 cases, 978 controls	yes	[29]	Genotyped for rs1258763 in the present study
		Mexican	156 cases, 337 controls	yes	[30]	
		Yemeni	231 cases, 422 controls	no	[31]	
replication II	trio	European (EuroCran)	600 trios	yes	[10]	[10] ^g^

^a^—The meta-analysis of all three study cohorts in the present study is referred to as “combined analysis”.

^b^—The Ludwig 2012 meta-analysis contained two analyses, i.e., meta_Euro_ (in which the Central European case-control cohort and the European trios were combined), and meta_all_ (which additionally included the Asian trios). Full information on these analyses at 15q13 are provided in S1 Dataset.

^c^—Number of individuals included in each study. For replication I, pre-genotyping numbers are provided here while post-genotyping data can be found in S2 Dataset.

^d^—References are provided separately for description of the samples and genotype data for rs1258763, respectively.

^e^—All individuals are drawn from the Bonn cohort. Individuals included in the Ludwig 2012 meta-analysis have not been included in the replication I study. Therefore, both study cohorts can be considered independently.

^f^—In the present study, the 15q13 region was imputed using genotypes from Ludwig et al 2012.

^g^—In the EuroCran study that was part of Mangold et al. 2010, 65 trios from the Bonn cohort were included. To avoid overlap of individuals in the combined analysis of the present study, these individuals were excluded and data re-analyzed. For further details, see sample description in the Methods section.

As part of the present study, rs1258763 was then genotyped in an independent case-control cohort of mixed ethnicity (replication I, **Table 1** and **S2 Dataset**). After quality control, 580 cases and 1,684 controls remained in the analysis. In this replication I data, rs1258763 showed strong association with nsCL/P (*P*_nsCL/P_rep_I_ = 4.34×10^−09^, relative risk (RR) for the major allele A: 1.58, 95% confidence interval (95% CI): 1.36–1.84, **S2 Dataset**). Additionally, rs1258763 had been previously genotyped in an independent trio sample from the EuroCran cohort [10] (replication II, **Table 1**). Re-analysis of this data (to exclude individuals that were also part of replication I) revealed *P*_nsCL/P_rep_II_ = 0.072 (**S2 Dataset**), with the A-allele being overtransmitted to affected children.

Combined analysis of the Ludwig 2012 meta-analysis and both replication I and II data sets, respectively, totaling 979 nsCL/P cases, 3,002 controls, and 2,061 trios, yielded genome-wide significance (*P*_nsCL/P_comb_ = 2.23×10^−13^), with a strong genetic risk observed for the major allele A (1.35 (1.24–1.46), **S2 Dataset**).

**Fig 2:**
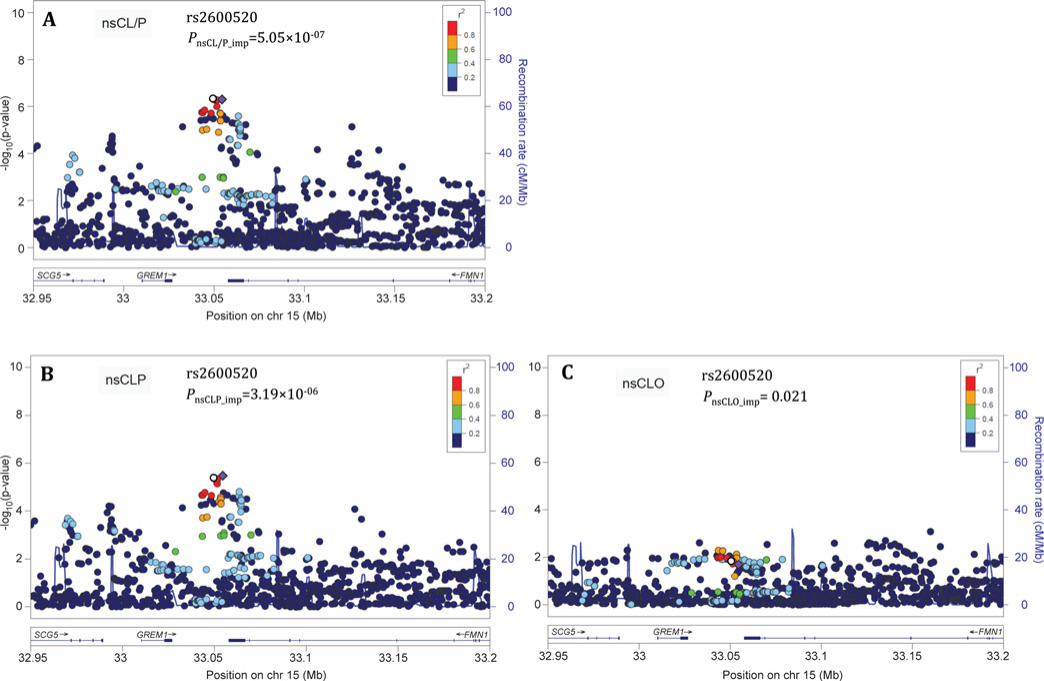
Regional association plots for the 15q13 region in different types of nsCL/P. In the imputed data of the Central European cohort, the 15q13 region was analyzed in the overall phenotype nsCL/P (**A**) and both subphenotypes, i.e. nsCLP (**B**) and nsCLO (**C**). For each SNP, the *P*-value is plotted against its chromosomal position (hg19). In nsCL/P and nsCLP, a highly associated cluster of SNPs in strong linkage disequilibrium is present, located between *GREM1* and *FMN1*. The lowest *P*-value was observed for rs2600520 (purple diamond). In each panel, the top genotyped variant rs1258763 is marked by an open circle. For all other variants, color code denotes linkage disequilibrium to rs2600520, based on 1000genomes. Regional association plots were generated using LocusZoom [28].

Imputation analysis of the 15q13 region in the Central European cohort that was part of the Ludwig 2012 meta-analysis (see Methods) revealed a 5 kb region of strongest association (Chr15: 33.050–33.055 Mb), located intergenically between *GREM1* and *Formin-1* (*FMN1*), with rs2600520 being the most strongly associated variant in the imputed data (*P*_nsCL/P_*_*_imp_ = 5.05×10^−07^, **Fig 2A** and **S3 Dataset**).

### Subphenotype analysis reveals strong association with cleft lip and palate

**Fig 3:**
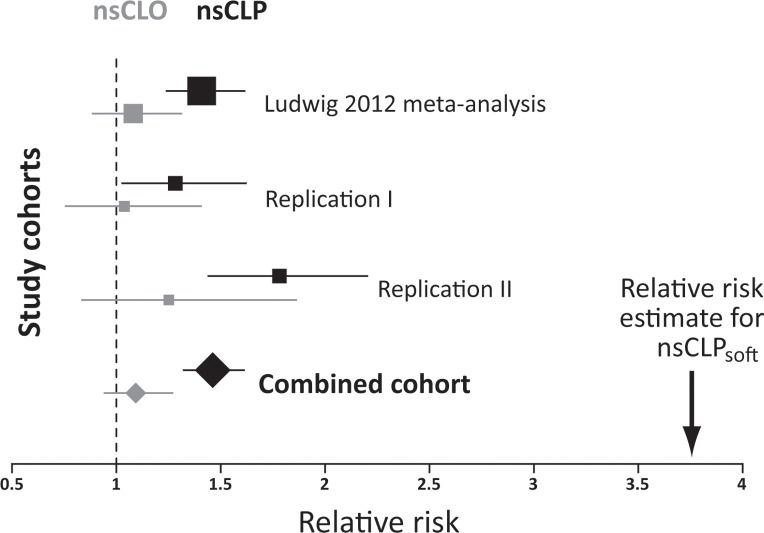
Forest plot for association of rs1258763 and nsCL/P subphenotypes. Subphenotype analyses of cleft lip and palate (nsCLP, black) and cleft lip only (nsCLO, grey) were conducted in the Ludwig 2012 meta-analysis data, the replication I and II cohorts, and in the combined analysis of the present study. Boxes represent point estimates of the relative risk for each of the four studies, with box sizes scaled according to the number of affected individuals. Lines indicate the extent of the confidence interval. These data illustrate the consistent association between rs1258763 and nsCLP across the various studies, and the presence of a narrow effect size range in the combined cohort. Please note that confidence intervals for nsCLO are larger due to the lower number of nsCLO patients. Informed by the specific expression of *Grem1* in lip and soft palate development in the mouse embryo (see below) we also analyzed the effect size of the particular soft palate subphenotype (nsCLP_soft_). The arrow indicates the point estimate for rs1258763 and nsCLP_soft_.

To determine associations between variants at 15q13 and particular clefting subphenotypes, individuals from the three study cohorts were classified as nsCLP or nsCLO on the basis of available clinical information (**S2 Dataset**). Variant rs1258763 showed significant association with nsCLP in the genotyped data of the Ludwig 2012 meta-analysis (*P*_nsCLP_meta_ = 8.0×10^−08^), replication I (*P*_nsCLP_rep_I_ = 1.28×10^−07^) and replication II (*P*_nsCLP_rep_II_ = 0.035 **Fig 3** and **S2 Dataset**). No significant association was detected for nsCLO (*P*>0.2 in all cohorts, **S2 Dataset**). For each of the three cohorts, the difference in relative risk between nsCLP and nsCLO was statistically significant (*P*<0.05).

The overall association *P*-value for nsCLP in the combined analysis was *P*_nsCLP_comb_ = 8.13×10^−14^ (1.46 (1.32–1.61), **Fig 3**), in contrast to *P*_nsCLO_comb_ = 0.27 (1.09 (0.94–1.27)) for nsCLO. The difference in relative risks between nsCLO and nsCLP in the combined sample was statistically significant in both multiplicative (*P* = 0.0015) and general (*P* = 0.0033) model. In the subphenotype analysis of the imputed data generated from the Central European case-control cohort rs2600520 remained the most significantly associated variant with nsCLP (*P*_nsCLP_imp_ = 3.19×10^−06^, **Fig 2B**), while only marginal associations were observed with nsCLO (**Fig 2C**).

### *In silico* annotation supports *GREM1* as candidate gene at 15q13

For the 5 kb region of strongest association, no compelling evidence for the presence of regulatory elements was found in ENCODE [32], except for a 200 bp region identified as DNAse hypersensitivity site in four cell types, three of which had been derived from skin tissue (chr15:33,052,666–33,052,875, hg19) [33]. In datasets of relevance to craniofacial development [34, 35], no active regulatory elements were detected.

According to HaploRegv3 [36], the top associated variant rs2600520 and a second single nucleotide polymorphism (SNP), rs2600519, which is in high linkage disequilibrium (LD) and located four base pairs away, modify a chromatin mark that involves the transcriptional repressor Sin3A. The analysis of blood-sample expression quantitative trait loci (eQTL) yielded 38 SNPs with cis-eQTL effects on *GREM1* expression, six of which had false discovery rates below 0.05 (**S1 Table**). The strongest eQTL effect was observed for rs17816375, which showed a *P*-value of 0.033 in the imputed nsCLP data. Of the 38 SNPs, the SNP with strongest association to nsCLP was rs16958561 (*P* = 2.82×10^−05^). This SNP maps around 6 kb distal to rs2600520, and the two SNPs are in substantial LD (D' = 1 in the European population (1000 Genomes phase 1)). No trans- or cis-eQTL effect was identified for *FMN1*, despite the fact that one *FMN1*-targeting probe was represented on the expression array used [37]. Analysis of chromatin interaction data [38] obtained in human epidermal keratinocytes (NHEK), umbilical vein endothelial cells (HUVEC) and mammary epithelial cells (HMEC) revealed a topologically associated domain (TAD) encompassing the entire *GREM1* gene, the associated region and parts of the *SCG5* and *FMN1* coding regions. Standard annotation of contact domains reveals a subdomain separating the 5' region of *FMN1* from its 3' end and *GREM1* (**S1 Fig**). Further analysis of the TAD data using virtual 4C reveals evidence that direct interaction loops are present between the associated region and the *GREM1* transcription start site at the given resolution (**S2 Fig**).

### *Grem1* is expressed in a specific pattern and alters palatal shelve morphogenesis *in vitro*

**Fig 4:**
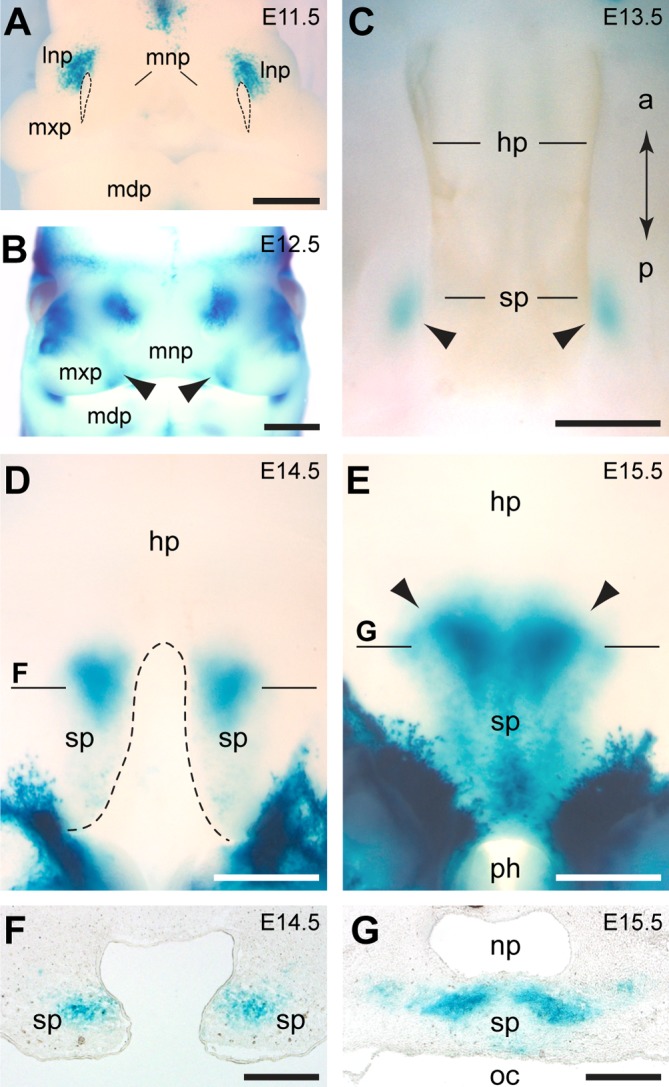
*Grem1* expression during mouse craniofacial development. (**A-E**) Expression of *Grem1* is visualized by X-Gal staining of heterozygous *Grem1*^*LacZ*^ whole mount embryos. (**A**) At E11.5, *Grem1* is expressed in the dorsal part of the lateral nasal prominence (lnp). Stippled lines demarcate the nasal pits. (**B**) At E12.5, *Grem1*-positive domains are also detectable in the merging zones (arrowheads) of medial nasal prominences (mnp) and maxillary prominences (mxp). (**C-G**) Secondary palate development. (**C**) At E13.5, *Grem1*-positive domains are observed in the forming soft palate (sp). (**D**) At E14.5, the hard palate (hp) has formed while the *Grem1*-expressing shelves of the soft palate are not yet fused. (**E**) At E15.5, the soft palate has fused and *Grem1* expression extends posterior to the pharynx (ph). Note the sharp anterior boundary of *Grem1* expression in the soft palate (arrowheads). (**F, G**) Sections of whole mount stained embryos. (**F**) Cross section at the level indicated in (D) showing that *Grem1* expression is restricted to the mesenchyme. (**G**) Cross section at the level indicated in (E) showing *Grem1* expression in the soft palate, which separates the nasopharynx (np) from the oral cavity (oc). Additional abbreviations: a, anterior; l, lateral; m, medial; mdp, mandibular prominence; p, posterior. Scale bars: 500μm.

X-Gal staining of the craniofacial region of heterozygous mutants of a mouse model of *Grem1* deficiency [17] revealed *Grem1* expression in the proximal region of both lateral and medial nasal prominences at E11.5 and E12.5 (**Fig 4A and 4B)**. In addition, bilateral *Grem1* expression was detected in the merging zones of the maxillary and medial nasal prominences during lip development at E12.5 (**Fig 4B**). *Grem1* was also expressed in the mesenchyme of the developing secondary palate between E12.5 and E15.5, with expression being restricted to the posterior palatal shelf region in which the soft palate forms (**Fig 4C–4G**).

**Fig 5:**
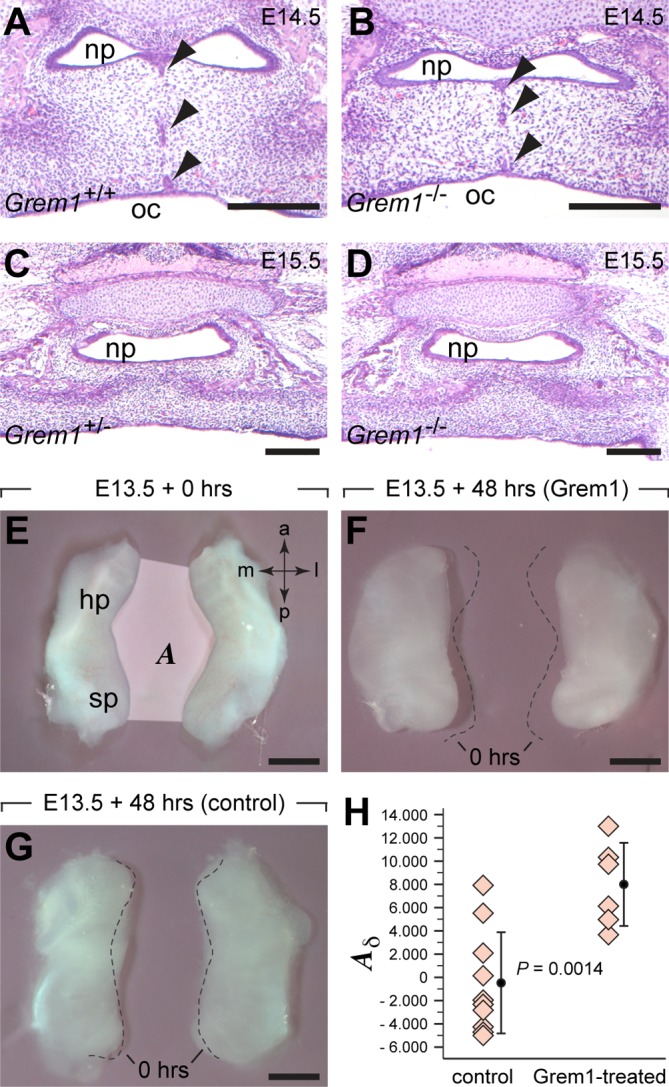
Analyses of the effect of *Grem1* loss of function and of ectopic Grem1 protein on secondary palate development. (**A-D**) Hematoxilin/Eosin staining of paraffin sections. (**A**,**B**) At the level of the posterior hard palate, the palatal shelves have fused and epithelial rests (arrowheads) are seen in both *Grem1*^+/+^ (**A**) and *Grem1*^-/-^(**B**) embryos at E14.5. At E15.5, the soft palate has fused in both *Grem1*^+/-^ (**C**) and *Grem1*^-/-^ embryos (**D**). (**E-H**) Organ culture experiments of secondary palatal shelves dissected at E13.5. (**E**) The area (*A*) between palatal shelves was measured at the onset and after 48 hours of culture. The presence of Grem1 protein led to an increase in the area (**F**), whereas the size of the area did not change in controls (**G**). Stippled lines demarcate the medial edges of the secondary shelves at the onset of culture. (**H**) The difference in area (*A*_δ_ **=** *A*_48hours_−*A*_0hours_) is significantly larger in the Grem1-treated palatal shelves compared to those of controls. Abbreviations: a, anterior; hp, hard palate; l, lateral; m, medial; np, nasopharynx; oc, oral cavity; p, posterior; sp, soft palate. Scale bars: 200μm in A-D, 500μm in E-G.

Although no craniofacial defects have been reported for *Grem1*-deficient mice [17], we tested the hypothesis that loss of *Grem1* results in alterations of lip and palate morphology without manifesting any profound craniofacial phenotype. However, a histological analysis revealed no developmental abnormalities in the embryonic secondary palate or during lip formation in *Grem1*^-/-^ mouse mutants (**Fig 5A–5D**). Next, E13.5 secondary palate shelves were cultured in the presence of exogenous Grem1 protein to assess whether this treatment could affect their development. Significant growth (approximately 25% increase) of the palatal shelves was observed in both Grem1-treated and control groups during the 48 hour culture period, with no significant difference in the final size of the shelves observed between groups (*P* = 0.27, **S2 Table**). However, Grem1-treated palatal shelves showed a more rounded shape, with retraction of their medial edges (**Fig 5E–5G**). Measurement of the area between both palatal shelves at 0 and 48 hours revealed a stable area in the control group but a significant increase in the Grem1-treated group (*P* = 0.0014 for difference between control and Grem1-treated pairs; **Fig 5H**, **S3 Table**).

### Genotype-phenotype correlation is strongest in a rare clinical entity

Informed by the murine expression pattern of *Grem1*, we tested for involvement of the soft palate in human data sets. Twenty-one patients with a cleft lip and a cleft of the soft palate in the presence of an intact hard palate (nsCLP_soft_) were selected (see Methods). In this group, analysis of rs1258763 revealed *P* = 0.03 and an RR of 3.76 (95% CI: 1.47–9.61) in nsCLP_soft_, representing a two-fold increase of effect size in comparison to 320 patients with complete cleft lip and palate (nsCLP_hard+soft,_ **Fig 3** and **Table 2**). While only a limited number of patients with this rare nsCLP_soft_ phenotype could be recruited this association *P*-value passed the statistical significance threshold after permutation-based correction (*P* = 0.04). No association was found in a group of 45 patients with cleft of the soft palate only and an intact lip (*P*_nsCPO_soft_ = 0.94, **Table 2**), or in 115 patients with a submucous cleft of the soft palate (*P*_nsCPO_submuc_ = 0.85, **Table 2**).

**Table 2:** Analysis of rs1258763-subphenotype effect on the soft palate.

Phenotype	Genotypes cases	MAF cases	Genotypes controls	MAF controls	*P-*value	RR allelic (95% CI)
nsCLP__hard+soft_	13/130/167	0.252	149/590/578	0.337	**3.53×10**^**−05**^	1.51 (1.24–1.85)
nsCLP__soft_	1/3/17	0.119			**2.99×10**^**−03**^	3.76 (1.47–9.61)
nsCPO__soft_	1/28/16	0.333			0.94	1.02 (0.65–1.59)
nsCPO__submuc_	12/55/48	0.343			0.85	0.97 (0.73–1.29)

Abbreviations: MAF—minor allele frequency, RR—allelic odds ratio (provided for the risk allele A), CI—confidence interval, ns—non-syndromic, CLP—cleft lip and palate, CPO—cleft palate only, submuc—submucous. Genotypes are presented with the minor allele first, i.e. GG/AG/AA.

## Discussion

Previous studies have reported suggestive associations between markers in the 15q13 region and nsCL/P [10, 15]. In the present study, we now conclusively confirm 15q13 as risk locus for nsCL/P by reaching genome-wide significance. In addition, the present study is the first to demonstrate a specific subphenotype effect in patients with nsCLP. While 15q13 is not associated with nsCLO in any cohort analyzed in this study, a strong association with nsCLP was observed in different ethnicities, suggesting the 15q13 risk locus generally acts on various population backgrounds. The strength of association, however, varied between ethnicities (1.28 to 1.87 in the present study), which might be due to heterogeneity at the population or allelic level, respectively. Notably, we observed the lowest effects size in the replication II cohort which is a trio data set of European ethnicity. As heterogeneity can be considered rather unlikely here, an additional explanation would be differences in the composition of the samples, e.g., in the frequency of individuals with a positive family history. This should be investigated in further studies.

The region of strongest association, which was identified by imputation of common variants in Central Europeans, encompasses several variants in high LD located within a 5 kb region, about 40 kb downstream of the *GREM1* transcription start site (TSS). Two hypotheses regarding the nature of the functional causative variant(s) arise: First, the common variant(s) might be functionally relevant themselves. This has been previously observed in nsCL/P for common variants at the high-risk locus *IRF6*, where a common variant in the *IRF6* enhancer mediates craniofacial disturbances by altering an Ap-2alpha binding site [5] and, recently, for rs227727 at the 17q22-locus [39]. Alternatively, rare but highly penetrant sequence variants might confer functional effects in some patients. Those variants are missed by imputation, and their detection would require re-sequencing of the entire associated region in large cohorts of either multiply affected families (to infer co-segregation) or trios with sporadic cases (to detect *de novo* occurrences). The successful outcome of such an approach has recently been demonstrated in a large trio re-sequencing study of nsCL/P risk loci. In that study, functionally relevant rare variants were identified, including a non-coding variant in *FGFR2* [39].

The formation of lip and palate is completed by the 10^th^ week of human embryonic development. In the absence of functionally annotated material from human embryonic craniofacial tissue we here used different approaches to assess a potential regulatory effect of the common variant rs2600520 or any other highly associated variants. First, we checked the functional regulatory landscape at 15q13 using previously published Hi-C data [38]. This analysis showed that *GREM1* locates within a topologically associated domain (TAD) that includes the associated region as well as parts of the adjacent genes *SCG5* and *FMN1*. Notably, *in silico* annotation of contact domains suggests that the 3' *FMN1* region, together with *GREM1*, is located in one functional unit which is different from that containing the *FMN1* 5' region and *FMN1* promoter. While closer analysis of the region reveals evidence for an interaction of the *GREM1*-TSS with the *GREM1*/*FMN1* intergenic region, little evidence is provided for interaction between the associated region and any of the *SCG5* or *FMN1* promoters.

We also accessed data from a large and systematic analysis of gene expression from blood samples [37], using *FMN1* and *GREM1* as query genes. While no result was returned for *FMN1*, numerous eQTLs for *GREM1* were identified. This suggests a regulatory effect of SNPs in this intergenic region on *GREM1* in general, however, none of the SNPs identified at an FDR < 5% are associated with nsCLP in our imputed data. Notably, two of the variants with suggestive evidence (rs16958561, rs16958734) are significantly associated in our imputed case-control data and are in high LD with rs2600520. This suggests that common functional variant(s) located on haplotypes tagged by rs2600520 could have an effect on *GREM1* expression and might be stronger eQTLs in data sets of relevant craniofacial tissue. Next, the 5 kb risk locus was analyzed in comprehensive data from the ENCODE project [32] and published datasets of relevance to craniofacial development [34, 35]. No compelling evidence for the presence of regulatory elements was found in these data, with the exception of a 200 bp DNA hypersensitivity site [33] in ENCODE, which indicates accessible chromatin in this region. Notably, the DNA region around rs2600520 and rs2600519 has previously been identified as chromatin mark involving the transcriptional repressor Sin3a [36]. Together with histone deacetylases (HDAC1 and HDAC2) Sin3a can form part of a repressor complex that is targeted to specific DNA regions by sequence-specific transcription factors [40]. Interestingly, at E10.5 of mouse development *Sin3a* is expressed in the developing limb buds and in craniofacial prominences in a pattern overlapping with that of *Grem1* (Emage: #2906, [41]). It is conceivable that a failure to recruit the transcriptional repressor Sin3a to this putative regulatory region could result in up-regulated *GREM1* expression, which in turn would affect lip and palate development by disrupting Bmp signaling. However, the identity of the DNA-binding transcription factors that recruit Sin3a and recognize target sites near or at rs2600520 and rs2600519 in craniofacial prominences remain to be identified.

The functional annotation approach used in this study has two limitations. First, most of the analyses in functional data sets are based on tissue types with no direct relevance for nsCL/P. However, given the lack of appropriate nsCL/P-relevant material at the moment, these data provide the opportunity to understand basic regulatory mechanisms that might be present at the *GREM1* locus. Second, methodological issues such as under-investigation of particular epigenetic marks or activity states (such as silencers) in the queried datasets might have been confounding factors in our analysis. To identify regulatory non-coding regions and to decipher how the intergenic *GREM1*/*FMN1* region interferes with normal craniofacial development, the generation of comprehensive data sets in relevant tissues is warranted which might be accomplished by consortia projects such as Facebase [42].

Our data suggest that intergenic variants located close to *FMN1* affect regulatory elements which are targeting the adjacent *GREM1* gene. In nsCL/P, such long-range effects of genetic variants on distally located genes have been previously suggested, e.g. at the 1p22 locus: Here, associated variants map intronically within the *ABCA4* gene while expression and mutation analyses suggest the adjacent *ARHGAP29* gene as causative gene [43]. However, experimental proof of this regulation has not yet been obtained. For the 15q13 locus, evidence for long-distance regulatory effects on *GREM1* is provided by studies in other mammals. Research in mice has shown that the 3’-region of the *Fmn1* gene is necessary for *cis*-regulation of *Grem1* transcription [44]. Remarkably, the developmental limb and kidney defects observed in *Grem1*^-/-^ mice are similar to those of *ld* (*limb deformity*) mutant mice [17, 44], which carry mutations in the *Fmn1* 3’-region. These findings suggest that the *Grem1*-loss of function mutation is allelic with *ld*, and that *Grem1* expression might be regulated by a global control region (GCR) located near or at the *Grem1*/*Fmn1* intergenic region [17, 44]. The activity and function of specific regions within the GCR, however, might be different, depending on the developmental processes or tissues [45].

Our study also provides first evidence for genetic components underlying a rare clinical entity, i.e., a cleft lip and a cleft soft palate in the presence of an intact hard palate. Cuddapah et al (2015) coined the phrase “interrupted clefting” to describe this phenotype [46] and challenged the classical view that cleft lip with or without cleft palate are always manifestations of a single etiological continuum. During mouse embryogenesis, highly localized expression of *Grem1* was observed in the soft palate and in the processes forming the lip, while no expression was detectable in the developing hard palate. These observations correspond well with the considerably increased risk we observed in patients presenting with "interrupted clefting". Thus, our data support a more complex situation as they suggest that a cleft palate may form independently of a cleft lip and that nsCLP can be caused by disruptions of genetic pathways that are required in both lip and palate development. Although intriguing, this observation was based on a low number of individuals only and, therefore, has to be confirmed in further independent samples for which detailed clinical information is available. Within this context, it is interesting to note that genotype-phenotype correlations in six previously reported nsCLP multiplex families with rare mutations in *GREM1* [16] revealed a correlation between the presence of these mutations and cleft soft palate status in 11 out of 13 affected individuals (**S3 Fig**). All variants were located in the 3' UTR, and the functional impact of the identified mutations has not been established. Importantly, it has been recently shown that rare variants in the 3' and 5' UTR regions might have a strong effect on disease risk, which might even outweigh the effect of rare coding mutations [47]. We hypothesize that rare *GREM1* mutations might have modifier effects, thereby influencing which of the palatal structures are affected in patients predisposed to nsCLP. This would contribute to the explanation why unaffected individuals in these families are mutation carriers. Although not fully conclusive yet, this observation should be followed up in the future, including experimental validation of the functional role of the non-coding mutations.

Given the critical requirement of Grem1 in kidney and limb formation during mouse embryogenesis [17, 18] it is unlikely that a ubiquitous downregulation of *GREM1* transcription during human embryogenesis would exclusively affect lip and palate development. Moreover, our mouse data indicate that the presence of *Grem1* is not essential for the development of the lip and palate. However, *Grem1* expression is required for limb bud development, where it maintains a positive Shh-Fgf feedback loop through restriction of Bmp signaling, which in turn is a negative regulator of Shh expression in the limb mesenchyme [17, 48]. In marked contrast, a previous study identified Bmp signaling as a positive regulator of both Shh expression in the palatal shelf epithelium and cell proliferation in the anterior palatal shelf mesenchyme [22]. These findings suggest that increased or ectopic expression of *Grem1* might interfere with normal secondary palate development through: (i) inhibition of Bmp4 or Bmp2, both of which are expressed in the anterior palate at E12.5 [22]; or (ii) inhibition of Bmp4 and Bmp7, both of which are strongly expressed in the posterior region of the palatal shelves at E13.5 in the mouse [49]. The results of our organ culture experiments suggest that a pathogenic effect can be caused by increased levels of Grem1, as these data demonstrated a marked effect of ectopic Grem1 protein on the morphogenesis of the embryonic palatal shelves during a critical phase of secondary palate development.

Furthermore, the nsCL/P risk allele for rs1258763 has been associated with increased nasal width [50, 51], which is consistent with results from a previous meta-analysis showing an increased nasal width in unaffected parents of nsCL/P patients [52]. This provides additional support for the hypothesis that regulatory regions near the *GREM1* locus plays an active role in modulating morphogenesis during craniofacial development.

Although our results support the hypothesis that *GREM1* is a candidate gene at 15q13 and expression analyses of various isoforms of murine *Fmn1* transcripts [53] failed to implicate *Fmn1* in lip and palate development, our data do not entirely exclude the possibility that *Fmn1* could play some role in craniofacial development. *Fmn1* has been shown to regulate aspects of mouse limb and kidney organogenesis similar to those controlled by *Grem1* [54]. However, contrary results in terms of expression regulation have been obtained. Whereas *Grem1* expression was upregulated in a *Fmn1* null mouse mutant in which the *Grem1*/*Fmn1* regulatory region was intact [54], earlier observations demonstrated reduced *Grem1* expression as a result of the *ld* mutation, which affects the 3’-region of *Fmn1* [44]. Thus, the *Grem1*/*Fmn1* regulatory landscape exhibits a complex architecture, which, at present, cannot be delineated more precisely due to the limited resolution of chromatin interaction data and the close proximity of the *GREM1* and *FMN1* genes. However, our results suggest strong, and functionally distinct regulatory activities in the region, which warrants investigation in future studies that might include higher chromatin resolution and targeted conformation assays.

### Concluding remarks

The present study established that the 15q13 region contains a genetic risk factor for nsCL/P. This is the second locus after 13q31 to show a particularly strong association with nsCLP and not with nsCLO. Moreover, our results suggest this risk factor to be involved in nsCLP_soft_, and our genetic and functional analyses provide strong support for the hypothesis that *GREM1* is an effector gene that contributes to this rare clinical subphenotype. In aggregate, our data suggest that *GREM1* plays a specific role not only in the development of the lip, but also during formation of the soft palate. These findings provide a framework for further functional analyses of *GREM1* in human cell systems and for inducible modulation of *Grem1* expression during the development of specific craniofacial regions in model organisms. These analyses may broaden our understanding of the processes that regulate facial morphogenesis, and help to decipher the molecular mechanisms underlying the manifestation of specific nsCL/P subphenotypes in humans.

## Materials and Methods

### Ethics statement

#### Human genetic studies

The study was approved by the ethics committees of the respective medical faculties, and informed consent was obtained from all participants.

#### Animal studies

Breeding and mouse embryo production were approved by the local veterinary authorities (permit 98/2011, Veterinäramt Zürich) in accordance with Swiss federal law (TSchG, TSchV) and cantonal by-laws in full compliance with European Guideline 86/609/EC.

### Sample description

#### Genome-wide cohorts

Association statistics for variants at 15q13 were obtained from a large nsCL/P meta-analysis performed by our group, which is described elsewhere [12] and referred to as “Ludwig 2012 meta-analysis” throughout this manuscript. This study included analyses of (i) European individuals (399 cases, 1.318 controls and 666 case-parent trios, referred to as meta_Euro_), and (ii) Asian and European individuals (inclusion of additional 795 Asian trios, referred to as meta_all_). Please note that the Central European cases from the case-control cohort were drawn from the same Bonn cohort as was part of the replication I sample. However, individuals were included either in the GWAS or in the replication; none of the individuals was included in both studies.

#### Replication cohorts

1. Replication I (case-control cohort). The first replication cohort was a case-control cohort that comprised nsCL/P samples from three different populations (Bonn, Mexico, Yemen). The Bonn sample comprised 223 independent nsCL/P patients. A total of 978 volunteer blood donors were included as controls, which should not result in any appreciable reduction in power given the low prevalence of nsCL/P in the general population [55]. The Mexican case-control sample was recruited as described elsewhere, and subphenotype information was available for these subjects [30]. The Yemeni case-control sample was recruited as described elsewhere [31]. No information on subphenotypes was available for the Yemeni sample.
2. Replication II (EuroCran trio cohort). Genotypes for individuals from the EuroCran cohort for rs1258763 were retrieved from a previously published study [10]. In this study from 2010, the EuroCran sample contained 65 trios that were from the Bonn cohort and therefore, some of the affected index patients overlapped with individuals from the replication I cohort. To avoid any overlap in the statistical analysis, we excluded these 65 Bonn trios from the EuroCran data and re-analyzed the nsCL/P association (replication II). This explains differences in T/NT ratios and *P*-values between the present study and Mangold et al 2010. Notably, in Mangold et al. 2010, no subphenotype analysis for rs1258763 was performed.

#### Analysis of clinical subtypes

To characterize the cleft lip and palate phenotype in more detail, all available clinical information was accessed. For individuals of the Central European case-control cohort from Mangold et al. 2010, phenotype information was retrieved from our in-house clinical database. A total number of 310 patients with a complete cleft of the lip and clefts of the hard and soft palates were identified (CLP_hard+soft_). Thirteen patients from the Bonn cohort (8 from the GWAS, 5 from the replication I cohort) displayed a cleft of the lip and the soft palate in the presence of an intact hard palate (CLP_soft_), indicating the rarity of this clinical subphenotype. To increase the size of this CLP_soft_ group we reached out to other clinical nsCL/P cohorts and identified eight patients meeting the CLP_soft_ criteria in a Dutch nsCL/P sample [56]: Six of these patients were part of the EuroCran cohort while two patients were drawn from an independent Dutch sample [57]. In addition, rs1258763 was genotyped in: (i) 45 patients with a cleft of the soft palate only (CPO_soft_), which is an orofacial clefting subform with a different genetic background; and (ii) 115 patients from a cohort of submucous cleft palate patients (CPO_submuc_) [58].

### Genotyping and statistical analysis

Genotyping of rs1258763 in the nsCL/P replication sample and nsCPO_soft_ individuals was performed using the Sequenom MassArray system (Agena Bioscience, San Diego, USA). nsCLP_soft_-patients from the Dutch cohort and the nsCPO_submuc_ patients were genotyped using ABI3130XL sequencing and BigDye v3.1.

The replication II sample was analyzed using the transmission disequilibrium test. In the replication I cohort, genotype frequencies in the cases and controls of each of the three subsamples (Bonn, Mexico, Yemen) were compared using the Cochran-Armitage trend test. To combine all results for rs1258763, effect estimates for the different studies were combined in an inverse variance-weighted fixed-effects meta-analysis. To test for heterogeneity of the genotypic RRs between the nsCLO and nsCLP phenotypes, a heterogeneity likelihood-ratio test was applied using a general and a multiplicative model. For each of these analyses, this resulted in an asymptotic Chi^2^ null distribution with two degrees of freedom.

For the analysis of rs1258763 in the nsCL/P subphenotypes nsCLP_hard+soft_, nsCLP_soft_, nsCPO_soft_, and nsCPO_submuc_, a common set of 1,318 controls from the Central European case-control cohort was used. To determine whether a statistically significant difference was present between the results for nsCLP_hard+soft_ and nsCLP_soft_ and to account for the limited number of individuals in the nsCLP_soft_ group, an empirical *P*-value was determined using 100,000 permutations (larger test statistics were assigned a value of 1, equal test statistics were assigned a value of 0.5).

### Imputation analysis

Genotypes from the Central European case-control cohort that was part of the meta-analysis [10] were imputed using IMPUTE2 [59] and 2,184 alleles of the 1000genomes project. Dosage values were included in a logistic regression model (using SNPTEST and—method expected) in which we included as covariates the first five components e_ik_ (which were obtained from MDS analysis [60], to account for population stratification. Only variants showing a SNPTEST info-score (detailed description: https://mathgen.stats.ox.ac.uk/genetics_software/snptest/snptest.v2.pdf) of at least 0.4 and a minor allele frequency of at least 1% were retained, as accuracy of imputation is compromised for low frequency variants. The resolution of the 15q13 region (250 kb, chr15:32.95 Mb—33.2 Mb) increased from 106 variants in the genotyped data to 1,042 variants in the imputed data. Relative risks were calculated using the beta values, representing the logarithm of the RR. Please note that inclusion of the genome-wide data from Beaty et al. 2010 in the imputation analysis was not possible at the time of the present study, due to insufficient coverage of that approach in the dbGaP approval used for the Ludwig 2012 meta-analysis.

### *In silico* annotation

Potential regulatory effects of the top associated region on either *GREM1* or *FMN1* were assessed using previously published eQTL and HiC-datasets. Since there is a complete absence of eQTL studies of fetal craniofacial samples, eQTLs obtained in large datasets from other tissues were used. Here we used a recently published and comprehensive study of blood samples by Westra et al. [37]. Both *FMN1* and *GREM1* probes are present on the Illumina arrays used in the Westra et al. study. To identify altered transcription factor binding we used the v3 version of the HaploReg tool, which was developed to annotate disease-associated genetic variants located in non-coding regions [36]. In particular, information on transcription factor binding altered by nsCL/P risk alleles was used. These analyses were complemented by recently published HiC-data illustrating chromatin formation and loops at 15q13 using the Juicebox tool [38] and virtual 4C as provided at http://promoter.bx.psu.edu/hi-c/virtual4c.php using the same data.

### Mouse husbandry and *Grem1* expression analysis

*Grem1*^*LacZ*^ mutant mice [17] were kept on a C57Bl/6 genetic background and embryos were staged using mid-day on the day of vaginal plug detection as embryonic day 0.5 (E0.5). Genotyping of embryos was carried out using allele-specific PCR. The wild type allele was detected as a 435bp product using primers 5’-TGCAATTGTGTCAGGAGCCA-3’ and 5’-ACTGGGTCTGCTCAGAGTCA-3’. The lacZ allele was detected as an approximately 550bp product using primers 5’-TGCAATTGTGTCAGGAGCCA-3’ and 5’-GGGAACAAACGGATTGACCG-3’. Following embryo dissection, mandibles and tongues were removed to facilitate the penetration of fixatives and staining solutions. Expression of *Grem1* was visualized by X-Gal staining of heterozygous *Grem1*^*LacZ*^ whole mount embryos using standard procedures, as described elsewhere [61]. To assess tissue-specific domains of *Grem1* expression, 15μm cryosections of whole mount stained embryos were prepared and mounted onto glass slides. Images were obtained using an Axiocam color camera mounted on a Zeiss Stemi SV11. Images were processed using the softwares Axiovision AC (release 4.4) and Photoshop C4 (version 11).

### Organ culture experiments and histology

Organ culture experiments of secondary palatal shelves were carried out using conditions essentially similar to those described elsewhere [61, 62]. Briefly, palatal shelves were dissected at E13.5 and cultured for 48 hours on sterilized filter papers (Millipore) placed on a metal grid located on the central well of a culture dish. With the aboral side facing downwards, the palatal shelves were cultured in Dulbecco’s modified eagles medium (DMEM), supplemented with 10% (v/v) fetal bovine serum, 1% (v/v) penicillin/streptomycin (5000 units/ml, 5000ug/ml), 1% glutamine (200mg/ml) and 1% (v/v) ascorbic acid (50mg/ml). Grem1 recombinant protein (Peprotech (USA)) was used at a final concentration of 10μg/ml medium. The organ rudiments were cultured at 37°C using 5% CO_2_ for 48 hours and culture medium was replaced after 24 hours. Images were obtained using a Axiocam mounted onto a Zeiss Stemi SV6 stereomicroscope. Areas between palatal shelves were measured using imagej (http://imagej.nih.gov/ij/). To calculate *P*-values, arbitrary units quantifying the area between the palatal shelves were analyzed using a one-tailed t-test. For the craniofacial phenotype analysis of *Grem1*-deficient mouse embryos, processing of heads for embedding in paraffin and generation of Hematoxilin/Eosin stained sections were performed as described elsewhere [61].

### URLs

For the purposes of the present study, the following databases were accessed: https://www.broadinstitute.org/mpg/snap/ [63], http://genenetwork.nl/bloodeqtlbrowser/ [37], http://www.emouseatlas.org/gxdb/dbImage/segment1/2906/2906.html [41], http://www.aidenlab.org/juicebox/ [38].

## Supporting Information

S1 Fig Chromatin interaction analyses data for the 15q13 locus. Chromatin interaction data for three different celltypes were drawn from [38]. RefSeq genes are plotted above the interaction map, together with CTCF binding sites identified in the respective cell lines. *GREM1* position is highlighted in red, adjacent genes *SCG5* and *FMN1* are also labeled. + /—below the RefSeq annotation denotes strand orientation of the gene. Yellow lines indicate contact regions, blue squares show regions of loop interactions, both as defined by the original study. The dotted line indicates co-localization of the *GREM1 / 3‘FMN1* region within one topologically associated domain (TAD) which is separate from the 5‘ *FMN1* region. TAD structure is stable between each of the three cell types. (**A**) NHEK–normal human epidermal keratinocytes, in situ combined, 5kb resolution, (**B**) HUVEC–human umbilical vein endothelial cells, in situ combined dataset, 5kb resolution(**C**) HMEC–human mammary epithelial cells.
(TIF)

S2 Fig Local chromatin structure at *GREM1* / *FMN1*. Zoom-in into the *GREM1*/*FMN1* region is provided for the HUVEC cells. (**A**) Local TAD structure (chr15: 33.000.000–33.100.000) with arrow indicating potential interaction loops between the *GREM1* transcription start site (TSS, 33,010,204) and the intergenic region (33,040,000–33,044,999). The exact location of the loop cannot be further narrowed down due to the given resolution (5 kb). Data were drawn from [38]. Color code denotes number of observed reads. (**B**) Same data in virtual 4C visualisation. Using the *GREM1*-TSS as anchor point, regions of interactions are provided as peaks, with number of observed read counts as quantitative measure. Again, arrow highlights a potential interaction candidate at 33,040,000–33,044,999 bp.
(TIF)

S3 Fig Pedigrees of families with an index patient carrying a rare *GREM1* mutation. Phenotype: empty symbol–unaffected, half-filled symbol–cleft of the lip and hard palate only (soft palate intact), full symbol–cleft of the lip, hard and soft palate. Carrier status: + carrier,—non-carrier. Phenotype-genotype correlation: red symbols—individuals with concordant genotype-phenotype correlation, black symbols–individuals with discordant genotype-phenotype correlation. ^a^—Family BN-45 has four variants that are transmitted together: rs2280738, rs117317622, rs137899769, rs151194761. Please note that four more index patients with rare mutations but without any additional affected family members have a complete cleft of lip, hard and soft palate: BN-139 (rs201006159), BN-241 (rs201134502), BN-251 (g.33023715), BN-317 (rs147141645). All positions hg19.
(TIF)

S1 Dataset Summary statistics for genotyped variants at 15q13. (XLS)

S2 Dataset Association results for rs1258763 in different study cohorts. (XLS)

S3 Dataset Summary statistics and imputation results for 15q13 in Central European case-control cohort. (XLS)

S1 Table Association results and in silico annotation (A) for all variants with P<10^−5^ in imputation analysis and (B) for 38 SNPs with eQTL effects on *GREM1*. (PDF)

S2 Table Sizes of E13.5 palatal shelves cultured in the absence or presence of recombinant Grem1 protein. (PDF)

S3 Table Ectopic Grem1 protein causes differences in the morphogenesis of cultured palatal shelves. (PDF)
