# Supplementary figures and images for: Meta-analysis Reveals Genome-Wide Significance at 15q13 for Nonsyndromic Clefting of Both the Lip and the Palate, and Functional Analyses Implicate GREM1 As a Plausible Causative Gene
Source: PLoS Genet. 2016 Mar 11;12(3):e1005914. doi: 10.1371/journal.pgen.1005914 (PMC4788144; doi:10.1371/journal.pgen.1005914)

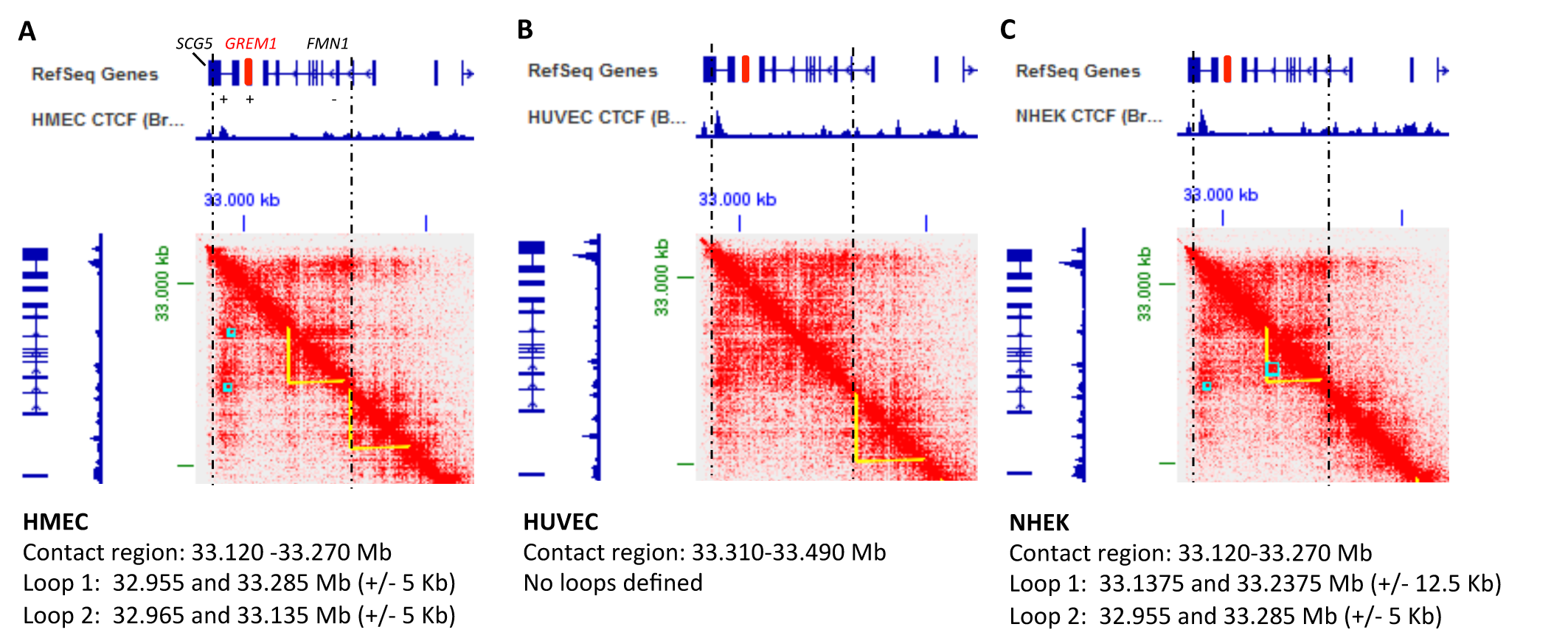

Supplement: S1 Fig — Chromatin interaction data for three different celltypes were drawn from [38]. RefSeq genes are plotted above the interaction map, together with CTCF binding sites identified in the respective cell lines. GREM1 position is highlighted in red, adjacent genes SCG5 and FMN1 are also labeled. + /—below the RefSeq annotation denotes strand orientation of the gene. Yellow lines indicate contact regions, blue squares show regions of loop interactions, both as defined by the original study. The dotted line indicates co-localization of the GREM1 / 3‘FMN1 region within one topologically associated domain (TAD) which is separate from the 5‘ FMN1 region. TAD structure is stable between each of the three cell types. (A) NHEK–normal human epidermal keratinocytes, in situ combined, 5kb resolution, (B) HUVEC–human umbilical vein endothelial cells, in situ combined dataset, 5kb resolution(C) HMEC–human mammary epithelial cells. (TIF) [file pgen.1005914.s001.tif]

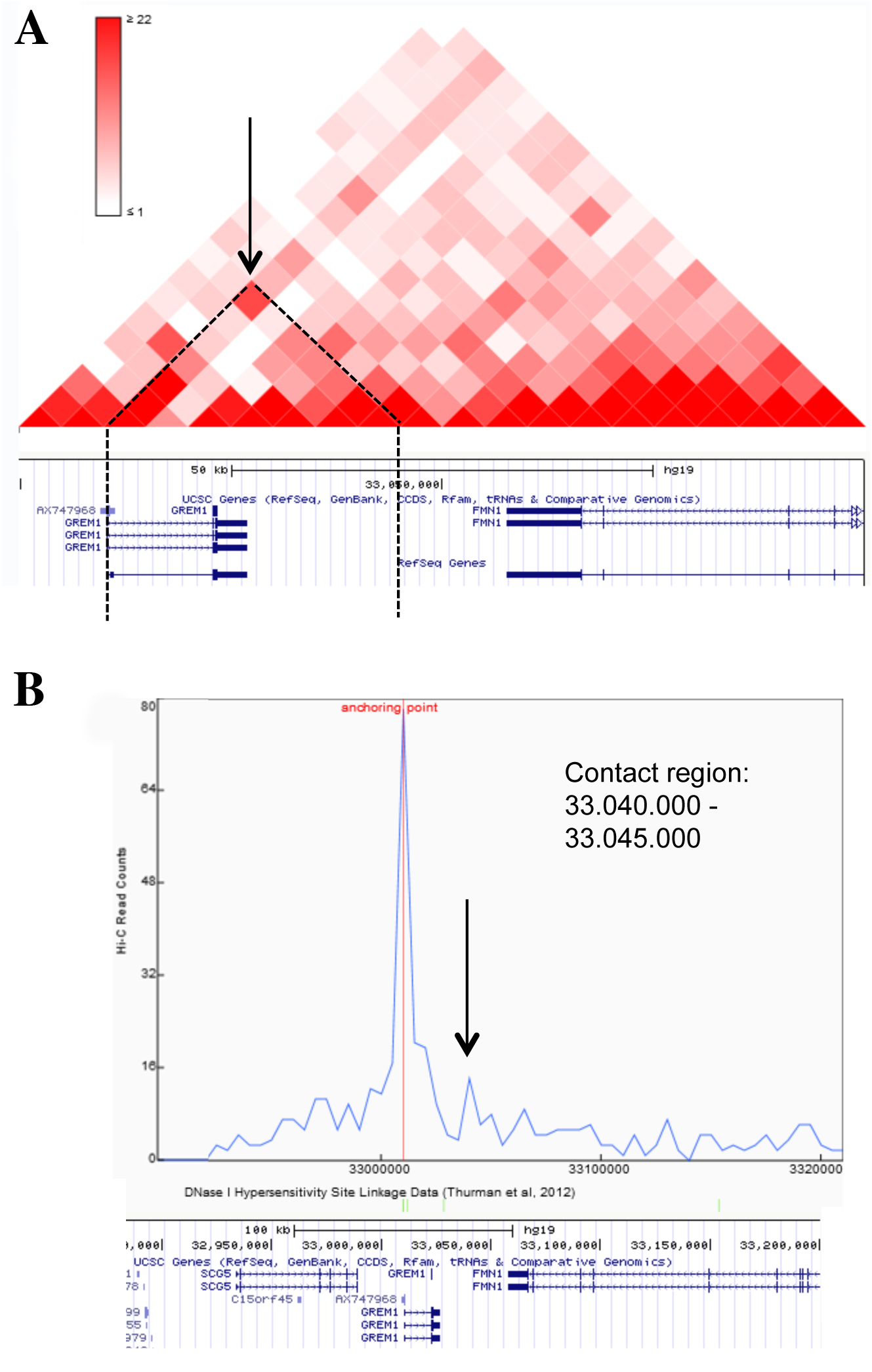

Supplement: S2 Fig — Zoom-in into the GREM1/FMN1 region is provided for the HUVEC cells. (A) Local TAD structure (chr15: 33.000.000–33.100.000) with arrow indicating potential interaction loops between the GREM1 transcription start site (TSS, 33,010,204) and the intergenic region (33,040,000–33,044,999). The exact location of the loop cannot be further narrowed down due to the given resolution (5 kb). Data were drawn from [38]. Color code denotes number of observed reads. (B) Same data in virtual 4C visualisation. Using the GREM1-TSS as anchor point, regions of interactions are provided as peaks, with number of observed read counts as quantitative measure. Again, arrow highlights a potential interaction candidate at 33,040,000–33,044,999 bp. (TIF) [file pgen.1005914.s002.tif]

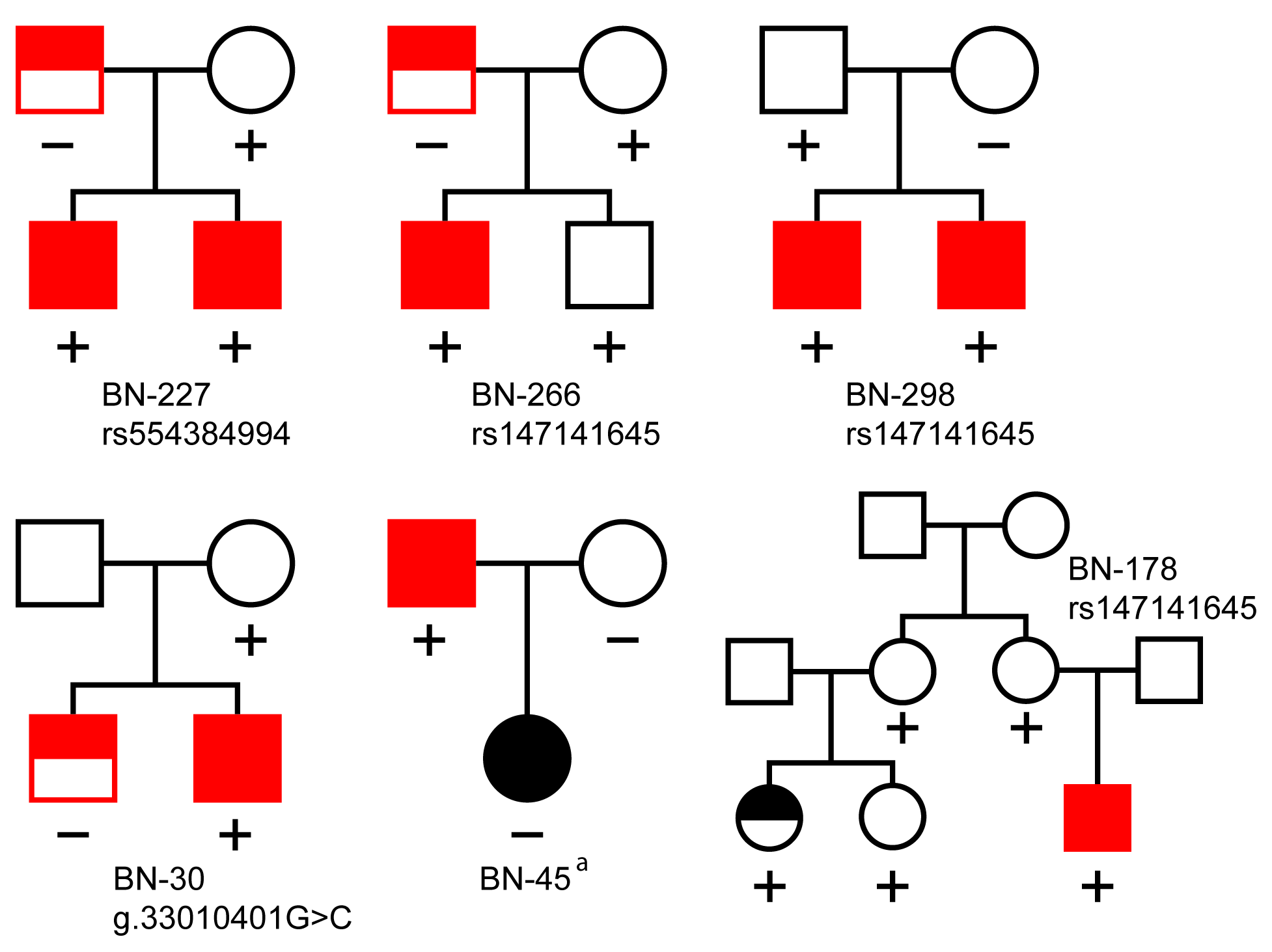

Supplement: S3 Fig — Phenotype: empty symbol–unaffected, half-filled symbol–cleft of the lip and hard palate only (soft palate intact), full symbol–cleft of the lip, hard and soft palate. Carrier status: + carrier,—non-carrier. Phenotype-genotype correlation: red symbols—individuals with concordant genotype-phenotype correlation, black symbols–individuals with discordant genotype-phenotype correlation. a—Family BN-45 has four variants that are transmitted together: rs2280738, rs117317622, rs137899769, rs151194761. Please note that four more index patients with rare mutations but without any additional affected family members have a complete cleft of lip, hard and soft palate: BN-139 (rs201006159), BN-241 (rs201134502), BN-251 (g.33023715), BN-317 (rs147141645). All positions hg19. (TIF) [file pgen.1005914.s003.tif]
