# Supplementary material for: Meta-analysis Reveals Genome-Wide Significance at 15q13 for Nonsyndromic Clefting of Both the Lip and the Palate, and Functional Analyses Implicate GREM1 As a Plausible Causative Gene
Source: PLoS Genet. 2016 Mar 11;12(3):e1005914. doi: 10.1371/journal.pgen.1005914 (PMC4788144; doi:10.1371/journal.pgen.1005914)
Supplement: S1 Table — (PDF) [file pgen.1005914.s007.pdf]

S1 Table: Association results and *in silico* annotation

(A) for all variants with $P<10^{-04}$ in imputation analysis of the nsCLP subphenotype

SNP	Chr.	Pos (hg19)	RA	RA freq. (%)	*P*-value	RR	lower RR	upper RR	info-score	cis-effect on *GREM1* in blood *	D' to rs2600520 in CEU	HaploReg motifchange by Ward and Kellis, 2012, Nuc Acid Res
rs2600520	15	33054519	G	67.13	**3.19E-06**	1.6	1.31	1.97	0.98	not reported	1	Egr-1_disc1;**Sin3Ak-20**_disc5;Zfx
rs2600519	15	33054523	A	66.94	**3.29E-06**	1.6	1.31	1.96	0.99	not reported	1	**Sin3Ak-20**_disc5;Zfx
rs1258763	15	33050423	T	66.27	**4.25E-06**	1.58	1.29	1.93	1.00	not reported	1	Cdx2_1;GR_known2;Hoxb13
rs1258757	15	33051684	C	66.38	**4.39E-06**	1.58	1.29	1.93	1.00	not reported	1	Pbx-1_4;RREB-1_1;TCF4_known1
rs1258756	15	33051810	G	66.38	**4.41E-06**	1.58	1.29	1.93	1.00	not reported	1	Mef2_disc1;Mef2_known2;Mef2_known3;Mef2_known6
rs1258762	15	33050657	T	66.37	**4.47E-06**	1.58	1.29	1.93	1.00	not reported	1	HDAC2_disc6;Pax-4_4;Sox_5;TATA_disc7;ZEB1_known2;p300_disc5
rs1258758	15	33051606	A	66.76	**7.03E-06**	1.57	1.28	1.92	0.99	not reported	1	ATF3_known6;Osr
rs3930082	15	33063527	C	44.45	**1.50E-05**	1.48	1.24	1.77	0.96	not reported	n.a.	AP-1_disc10;BCL_disc10;BCL_disc8;Egr-1_disc1;Egr-1_known1;Egr-1_known2;Egr-1_known3;Egr-1_known5;MOVO-B;Nrf1_disc3;PLAG1;Sp4;TCF12_disc3
rs9635315	15	33044871	G	70.44	**1.67E-05**	1.57	1.27	1.95	0.99	not reported	1	Mef2_known5;RXRA_known5
chr15:32993757:I	15	32993757	GC	6.94	**1.75E-05**	2.3	1.59	3.33	0.60	not reported	n.a.	n.a.
rs7179954	15	33054914	A	20.48	**1.75E-05**	1.56	1.28	1.91	0.98	not reported	n.a.	Evi-1_2
chr15:32993756:I	15	32993756	CC	6.9	**1.83E-05**	2.3	1.59	3.32	0.60	not reported	n.a.	n.a.
rs17816327	15	33056943	G	16.7	**2.09E-05**	1.58	1.28	1.94	1.00	not reported	1	CEBPB_known6;p300_disc2
rs11857585	15	33043284	T	70.33	**2.10E-05**	1.56	1.26	1.93	1.00	not reported	1	Ets_known8;Foxa_disc5;NERF1a;YY1_known5
chr15:33071480:D	15	33071480	G	16.63	**2.12E-05**	1.59	1.29	1.96	0.98	not reported	n.a.	n.a.
rs7166046	15	33043835	C	70.33	**2.14E-05**	1.56	1.26	1.93	1.00	not reported	1	EWSR1-FLI1;Elf3;Foxa_known4;GATA_disc3;HDAC2_disc6;Nanog_disc2;TATA_disc7

rs11854905	15	33048409	A	70.35	**2.21E-05**	1.56	1.26	1.93	1.00	not reported	1	AP-1_disc10;CCNT2_disc2;CHD2_disc3;CTCF_disc5;E2F_disc3;E2F_disc7;Ets_disc9;GR_disc6;Irf_disc4;Irf_disc5;MZF1::1-4_1;MZF1::1-4_2;Myc_disc10;Myf_1;Nrf1_disc3;PU.1_disc3;Pax-4_5;Pax-5_known1;Pou2f2_disc2;Rad21_disc10;Rad21_disc7;SMC3_disc3;SP1_disc3;SP1_known1;SP1_known2;SP1_known4;STAT_disc7;Sp4;TATA_disc4;YY1_disc3;Zfp281
rs1979170	15	33063372	T	44.1	**2.35E-05**	1.46	1.22	1.75	0.97	not reported	n.a.	BDP1_disc1;CTCF_disc8;Ets_disc7;Irf_disc4;Irf_disc5;Myf_1;NRSF_disc5;RXRA_disc2;RXRA_known3;Rad21_disc1;Rad21_disc7;SMC3_disc1;SP1_disc3;Znf143_disc4
rs17816321	15	33053948	C	16.22	**2.56E-05**	1.59	1.29	1.96	0.98	not reported	1	BCL_disc4;Irf_disc3;Irf_known9;PU.1_disc2;Pax-5_disc3;RXRA_disc4;STAT_disc3;p300_disc5
rs2600527	15	33053500	T	64.26	**2.61E-05**	1.51	1.24	1.84	0.98	not reported	1	PPAR_2;SP1_known4
rs2697936	15	33053402	G	64.25	**2.62E-05**	1.51	1.24	1.84	0.98	not reported	1	.
rs2840190	15	33060407	A	16.77	**2.79E-05**	1.57	1.27	1.93	1.00	0.003448992	0.856	Rad21_disc9
rs16958561	15	33060040	C	16.77	**2.82E-05**	1.57	1.27	1.93	1.00	0.002830226	1	HDAC2_disc5;PRDM1_disc1;PRDM1_known1
rs28425489	15	33064556	A	43.05	**2.91E-05**	1.45	1.22	1.73	0.99	not reported	1	NF-I_1
rs10519742	15	33061723	G	16.8	**2.96E-05**	1.57	1.27	1.92	1.00	not reported	1	Hoxa5_1;Pou2f2_known3;Pou3f2_2
rs34362234	15	33053599	T	64.34	**3.04E-05**	1.51	1.24	1.84	0.97	not reported	1	Irf_known10;Osr
rs75144062	15	33062886	T	16.82	**3.04E-05**	1.56	1.27	1.92	1.00	not reported	1	.
rs11630940	15	33067592	A	16.85	**3.15E-05**	1.56	1.27	1.92	0.99	not reported	1	ATF3_known4;Cdx2_2;FXR_3;Hoxc10;Hoxc9;Pax-2_2
rs11633236	15	33047856	G	15.82	**3.32E-05**	1.58	1.28	1.96	0.99	not reported	1	CEBPB_known6;CEBPG;Hdx;STAT_disc4
rs28696324	15	33064654	C	42.96	**3.53E-05**	1.44	1.21	1.72	0.99	not reported	1	Cart1;HMG-IY_2;HNF1_7;Hoxd8;Ncx_2;Nkx2_11;STAT_known3;Zfp105
rs28403314	15	33064636	A	44.73	**3.56E-05**	1.46	1.22	1.74	0.94	not reported	1	.
rs28756786	15	33064574	A	42.98	**3.58E-05**	1.44	1.21	1.72	0.99	not reported	1	Gfi1b;Hoxa10;Maf_disc2;Maf_known3;Maf_known4
rs28379322	15	33064577	A	42.98	**3.58E-05**	1.44	1.21	1.72	0.99	not reported	0.93	Hoxa10
rs13329310	15	33052553	T	75.59	**3.74E-05**	1.61	1.28	2.04	0.92	not reported	n.a.	Mef2_known5

rs12050761	15	33063603	C	43.16	**4.33E-05**	1.44	1.21	1.71	1.00	not reported	1	.
rs3743106	15	33063809	T	43.06	**4.47E-05**	1.43	1.21	1.71	0.99	not reported	1	Smad_2
rs3825862	15	33064148	T	43.2	**4.57E-05**	1.43	1.2	1.71	1.00	not reported	1	ATF4;Bach2;p300_disc7
rs2600525	15	33053603	T	64.72	**4.58E-05**	1.5	1.23	1.82	0.97	not reported	1	Foxc1_1;Foxj2_1;Osr
rs11638456	15	33049662	G	16	**4.62E-05**	1.57	1.27	1.94	0.99	not reported	n.a.	.
rs2600524	15	33053641	A	64.76	**4.87E-05**	1.49	1.23	1.82	0.97	not reported	1	Nrf1_known2;Pdx1_2;Pou3f2_3
rs2600526	15	33053594	C	64.76	**4.89E-05**	1.49	1.23	1.82	0.97	not reported	1	Osr
rs28728267	15	33064474	G	43.23	**4.89E-05**	1.43	1.2	1.7	1.00	not reported	1	.
rs12915645	15	33045226	T	15.97	**5.21E-05**	1.56	1.26	1.93	0.99	not reported	1	GATA_known13;PLZF;Pou3f2_3;SRF_known3;SRF_known6
rs12904470	15	33042833	G	15.96	**5.61E-05**	1.56	1.26	1.93	0.99	not reported	1	Ascl2;DMRT1;E2A_2;E2A_5;HEN1_1;HEN1_2;Lmo2-complex_1;NRSF_disc1;NRSF_disc5;NRSF_known3;Pbx3_known1;Sin3Ak-20_disc1
rs28399071	15	32992658	G	88.95	**6.05E-05**	1.97	1.38	2.81	0.97	not reported	0.383	n.a.
rs28414020	15	33064461	C	43.46	**6.07E-05**	1.43	1.2	1.7	0.99	not reported	0.93	Foxo_3;Mef2_known2;Mef2_known5;TCF12_disc2
rs28630996	15	32993860	A	88.84	**6.37E-05**	1.95	1.37	2.77	0.98	not reported	0.599	n.a.
rs16969681	15	32993111	C	90.14	**6.84E-05**	2.05	1.4	2.99	0.98	not reported	0.493	n.a.
rs76173415	15	33032639	T	15.66	**7.17E-05**	1.56	1.26	1.93	0.99	not reported	0.827	GR_known2
chr15:32993754:I	15	32993754	TG	7.38	**8.00E-05**	2.12	1.47	3.04	0.61	not reported	n.a.	n.a.
rs16958734	15	33067145	C	17.3	**8.08E-05**	1.52	1.24	1.87	1.00	0.003426893	1	ATF3_known8;BDP1_disc1;Pou1f1_2
rs72717674	15	33126395	G	9.88	**8.28E-05**	1.81	1.36	2.41	0.76	not reported	0.66	n.a.

Chr. - chromosome, Pos - position, RA - risk allele, RA freq - frequency of the risk allele in percent in controls, RR - relative risk, CEU - Central European population. * Data drawn from Westra et al., 2013 [37]

(B) 38 SNPs with eQTL effect

SNP	Chr.	Pos (hg19)	RA	RA freq. (%)	*P*-value	RR	lower RR	upper RR	info-score	cis-effect on *GREM1* in blood*	D' to rs2600520 in CEU
**rs17816375**	15	33080747	C	28.43	3.29E-02	1.23	1.02	1.48	0.95	1.64E-05	0.21
**rs11632517**	15	33081949	C	28.42	3.16E-02	1.23	1.02	1.48	0.95	1.94E-05	0.21
**rs11631485**	15	33076037	C	28.56	3.45E-02	1.22	1.02	1.48	0.96	2.85E-05	0.10
**rs16958843**	15	33075645	C	28.52	3.45E-02	1.22	1.02	1.48	0.96	2.89E-05	0.10
**rs1258790**	15	33110065	T	46.37	2.15E-01	1.12	0.94	1.32	0.99	9.16E-05	0.02
**rs896505**	15	33126392	C	36.49	2.50E-01	1.11	0.93	1.33	0.97	9.75E-05	0.20
rs17228873	15	33145511	T	22.07	7.29E-01	1.04	0.84	1.28	0.99	1.47E-04	0.03
rs1258734	15	33067656	G	31.98	6.15E-02	1.19	0.99	1.42	1.00	1.48E-04	0.18
rs17228850	15	33138088	A	22.02	7.18E-01	1.04	0.84	1.28	0.98	1.62E-04	0.09
rs10519756	15	33148207	T	22.12	7.27E-01	1.04	0.84	1.28	1.00	1.68E-04	0.09
rs1258797	15	33106411	C	27.09	2.96E-01	1.11	0.92	1.33	0.99	1.71E-04	0.56
rs1534596	15	33114882	C	36.76	3.06E-01	1.1	0.92	1.31	0.96	3.05E-04	0.25
rs4780052	15	33154851	C	50.11	5.26E-02	1.19	1	1.42	1.00	4.69E-04	0.01
rs1258766	15	33129571	G	48.68	2.86E-01	1.1	0.92	1.3	0.99	7.67E-04	0.02
rs4780042	15	33129380	C	25.11	2.10E-01	1.13	0.93	1.36	1.00	8.20E-04	0.39
rs1258769	15	33123153	A	48.22	2.28E-01	1.11	0.94	1.32	0.97	9.76E-04	0.08
rs8035260	15	33158164	C	43.17	3.23E-02	1.21	1.02	1.45	0.97	1.11E-03	0.04
rs1258770	15	33118446	C	48.4	2.58E-01	1.1	0.93	1.31	0.99	1.33E-03	0.03
rs17816465	15	33156386	A	20	8.22E-01	1.03	0.82	1.28	0.92	1.58E-03	0.08
rs1020561	15	33060601	A	20.49	9.40E-04	1.41	1.15	1.72	1.00	1.65E-03	0.53
rs6494699	15	33101626	T	19.15	2.65E-02	1.28	1.03	1.6	0.90	2.20E-03	0.60
rs1258724	15	33061195	C	25.86	3.22E-01	1.1	0.91	1.34	1.00	2.21E-03	1.00
rs1258771	15	33116348	G	47.83	2.31E-01	1.11	0.93	1.32	0.98	2.47E-03	0.03
rs17816423	15	33121278	G	36.31	4.87E-01	1.07	0.89	1.28	0.97	2.47E-03	0.37
rs12101843	15	33124275	C	38.3	3.68E-01	1.09	0.91	1.3	0.98	2.61E-03	0.37

rs16958561	15	33060040	C	16.77	2.82E-05	1.57	1.27	1.93	1.00	2.83E-03	1.00
rs7178265	15	33105403	G	25	1.19E-01	1.16	0.96	1.4	1.00	3.09E-03	0.47
rs1258725	15	33061759	A	20.59	1.16E-03	1.4	1.14	1.7	1.00	3.20E-03	0.51
rs3110558	15	33062339	A	20.59	1.16E-03	1.4	1.14	1.7	1.00	3.20E-03	0.51
rs1258728	15	33062103	T	21.17	1.37E-03	1.39	1.14	1.7	0.98	3.20E-03	0.51
rs1258731	15	33064791	A	25.91	3.37E-01	1.1	0.91	1.33	1.00	3.20E-03	1.00
rs1258721	15	33057357	A	25.82	3.12E-01	1.1	0.91	1.34	1.00	3.26E-03	1.00
rs1258718	15	33078376	C	25.75	3.72E-01	1.09	0.9	1.33	0.96	3.27E-03	1.00
rs1057473	15	33063930	A	25.91	3.37E-01	1.1	0.91	1.33	1.00	3.35E-03	1.00
rs2600522	15	33054126	T	47.77	6.06E-01	1.05	0.88	1.24	0.98	3.35E-03	1.00
rs16958734	15	33067145	C	17.3	8.08E-05	1.52	1.24	1.87	1.00	3.43E-03	1.00
rs2840190	15	33060407	A	16.77	2.79E-05	1.57	1.27	1.93	1.00	3.45E-03	0.86
rs1258719	15	33078951	G	26.2	4.17E-01	1.08	0.89	1.32	0.94	3.48E-03	1.00

Chr. - chromosome, Pos - position, RA - risk allele, RA freq - frequency of the risk allele in percent in controls, RR - relative risk, CEU - Central European population. Six SNPs highlighted in bold have been detected as eQTL with a false discovery rate below 0.05. SNPs shaded in blue indicate potential causative variants with eQTL effect, lower risk allele frequency as the rs2600520 risk allele and linkage disequilibrium D’=1 compared to rs2600520. * Data drawn from Westra et al., 2013 [37].
