# Supplementary material for: Meta-analysis Reveals Genome-Wide Significance at 15q13 for Nonsyndromic Clefting of Both the Lip and the Palate, and Functional Analyses Implicate GREM1 As a Plausible Causative Gene
Source: PLoS Genet. 2016 Mar 11;12(3):e1005914. doi: 10.1371/journal.pgen.1005914 (PMC4788144; doi:10.1371/journal.pgen.1005914)
Supplement: S2 Table — (PDF) [file pgen.1005914.s008.pdf]

S2 Table: Sizes of E13.5 palatal shelves cultured in the absence or presence of recombinant Grem1 protein.

Arbitrary area units measured with imagej (http://imagej.nih.gov/ij/) are listed. Growth of palatal shelves was observed in both groups (control; N = 20, Grem1-treated; N = 12), however, neither final sizes nor the increase in size of the Grem1-treated palatal shelves differed from those calculated for the control group.

**Control**					**Grem1-treated**				
Sample	0 hours	24 hours	48 hours	% (48hours)	Sample	0 hours	24 hours	48 hours	% (48 hours)
1	39383	48299	56802	144.2297	1	29046	33699	37291	128.386
2	25522	27400	33545	131.4356	2	30246	29180	35459	117.2353
3	35017	32270	38641	110.3493	3	33876	32130	35737	105.4936
4	38444	41640	49537	128.855	4	31912	32214	41169	129.0079
5	33741	34218	40126	118.9236	5	40902	38121	46481	113.6399
6	28585	28468	37963	132.8074	6	28382	27229	37088	130.6744
7	23835	31008	41310	173.3166	7	24258	24458	32017	131.9853
8	29402	29000	34181	116.254	8	23336	25200	34882	149.4772
9	36958	33625	42328	114.53	9	29235	25952	30673	104.9188
10	33881	33709	44409	131.0735	10	28440	33898	39785	139.891
11	34576	39244	45108	130.4604	11	34633	28066	40921	118.1561
12	25912	24315	32254	124.4751	12	26704	33468	46114	172.6857
13	30512	28469	35393	115.997				Average	128.4626
14	34073	30133	39399	115.6311				**0 hours vs 48 hours; *P* =**	**5.86E-05**
15	33278	31203	38948	117.0383					
16	35007	38046	46145	131.8165					
17	27265	24819	31362	115.0266				**Size (48hours) Control vs Grem1-treated; *P* =**	**0.142536**
18	30939	26836	32743	105.8308				**Growth (%) Control vs Grem1-treated; *P* =**	**0.269946**
19	44493	36417	44952	101.0316					
20	32555	29802	41742	128.2199					
			Average	124.3651					
			**0 hours vs 48 hours; *P* =**	**1.74E-07**					
