# Supplementary material for: Meta-analysis Reveals Genome-Wide Significance at 15q13 for Nonsyndromic Clefting of Both the Lip and the Palate, and Functional Analyses Implicate GREM1 As a Plausible Causative Gene
Source: PLoS Genet. 2016 Mar 11;12(3):e1005914. doi: 10.1371/journal.pgen.1005914 (PMC4788144; doi:10.1371/journal.pgen.1005914)
Supplement: S3 Table — (PDF) [file pgen.1005914.s009.pdf]

S3 Table: Ectopic Grem1 protein causes differences in the morphogenesis of cultured palatal shelves.

Areas enclosed by 10 pairs (control) and 6 pairs (Grem1-treated) of palatal shelves were measured using imagej (http://imagej.nih.gov/ij/) at 0 hours (*A*), 24 hours and 48 hours (*A'*), respectively. The differences between A and A' (δ*A*) were not significant in the controls but the areas increased significantly in the Grem1-treated samples ($P = 0.00136$). A comparison between δ*A* of controls and δ*A* of Grem1-treated samples revealed a significant difference ($P = 0.00142$).

**Control**				**Grem1-treated**			
	0 hours	48 hours			0 hours	48 hours	
Sample	A	A'	δA(A'-A)	Sample	A	A'	δA(A'-A)
1	11687	7512	-4175	1	11129	14905	3776
2	11028	8967	-2061	2	12345	18431	6086
3	17320	15071	-2249	3	10996	21259	10263
4	14192	9488	-4704	4	23362	36290	12928
5	31420	26432	-4988	5	27414	37293	9879
6	30256	27533	-2723	6	31113	36136	5023
7	22689	24721	2032			Average	7992.5
8	25107	33106	7999			STDev	3558.22
9	36827	36921	94				
10	25796	31450	5654				
		Average	-512.1			**Control A vs A'; *P* =**	**0.36231**
		STDev	4455.03			**Grem1-treated A vs A'; *P* =**	**0.00136**
						**Control δA vs Grem1-treated δA; *P* =**	**0.00142**
